# Integrin mediates cell entry of the SARS-CoV-2 virus independent of cellular receptor ACE2

**DOI:** 10.1016/j.jbc.2022.101710

**Published:** 2022-02-10

**Authors:** Jiamnin Liu, Fan Lu, Yinghua Chen, Edward Plow, Jun Qin

**Affiliations:** 1 Department of Cardiovascular & Metabolic Sciences, Lerner Research Institute, Cleveland Clinic, Cleveland, Ohio, USA; 2 Department of Biochemistry, Case Western Reserve University, Cleveland, Ohio, USA; 3 Department of Physiology and Biophysics, Case Western Reserve University, Cleveland, Ohio, USA

**Keywords:** virus infection, SARS-CoV-2, integrin, COVID-19, ACE2, angiotensin-converting enzyme 2, COVID-19, coronavirus disease 2019, Cy5-RBD, cyanine5-labeled RBD, hACE2, human ACE2, HBSS, Hanks' banlanced salt solution, RBD, receptor binding domain, R^403^GD, arginine–glycine–aspartic acid, SARS-CoV-2, severe acute respiratory syndrome coronavirus 2, SPR, surface plasmon resonance

## Abstract

Coronavirus disease 2019 (COVID-19) is a highly contagious disease caused by severe acute respiratory syndrome coronavirus 2 (SARS-CoV-2). It is broadly accepted that SARS-CoV-2 utilizes its spike protein to recognize the extracellular domain of angiotensin-converting enzyme 2 (ACE2) to enter cells for viral infection. However, other mechanisms of SARS-CoV-2 cell entry may occur. We show quantitatively that the SARS-CoV-2 spike protein also binds to the extracellular domain of broadly expressed integrin α5β1 with an affinity comparable to that of SARS-CoV-2 binding to ACE2. More importantly, we provide direct evidence that such binding promotes the internalization of SARS-CoV-2 into non-ACE2 cells in a manner critically dependent upon the activation of the integrin. Our data demonstrate an alternative pathway for the cell entry of SARS-CoV-2, suggesting that upon initial ACE2-mediated invasion of the virus in the respiratory system, which is known to trigger an immune response and secretion of cytokines to activate integrin, the integrin-mediated cell invasion of SARS-CoV-2 into the respiratory system and other organs becomes effective, thereby promoting further infection and progression of COVID-19.

The coronavirus disease 2019 (COVID-19)–induced global pandemic has been ongoing for nearly 2 years causing >350 million infections. Although the massive vaccination effort at global scale has reduced the severe threat of the COVID-19 pandemic, the mortality rate remains still relatively high (from ∼5% in the beginning of pandemic to ∼2% now) with a total of >5.5 million deaths. The pathogen causing this disastrous disease is severe acute respiratory syndrome coronavirus 2 (SARS-CoV-2) that belongs to the family of coronaviruses including 229E, NL63, OC43, HKU1, MERS-CoV, and highly homologous SARS-CoV (1). However, SARS-CoV-2 is apparently much more contagious than any other coronaviruses, and the mechanism underlying its infectivity is not well understood. Each SARS-CoV-2 is ∼50 to 200 nm in diameter containing an RNA segment of 30,000 bases encoding the virus, four structural proteins, the spike, envelop, membrane, and nucleocapsid proteins. The nucleocapsid protein holds the RNA genome, whereas other proteins form the viral envelope (2). The spike protein contains 1273 amino acids that are divided into S1 domain (1–541), a linker (542–685), S2 domain (686–1213), a single-pass transmembrane segment (1214–1236), and a short cytoplasmic tail (1237–1273) (3, 4). Importantly, S1 utilizes a fragment (333–529) called its receptor binding domain (RBD) to recognize angiotensin-converting enzyme 2 (ACE2)—a minor variant of ACE (5) to initiate virus entry and infection (2, 4). Such ACE2-mediated virus entry was first found for the homologous SARS-CoV in 2003 (6), although SARS-CoV only caused ∼8000 infections and did not lead to global pandemic. ACE2 contains an N-terminal peptidase domain and a C-terminal collectrin renal amino acid transporter domain on its extracellular region, followed by a single-pass transmembrane domain and a small intracellular domain (4). ACE2’s major physiological role is to lower blood pressure by using the peptidase domain to hydrolyze angiotensin II (a vasoconstrictor hormone peptide) into angiotensin 1 to 7 (a vasodilator). The SARS-CoV-2 S1 RBD binding to ACE2 does not affect the activity of ACE2 but rather promotes the attachment of the virus onto the cell surface. The virus attachment is followed by a furin-mediated cleavage at its S1/S2 site to facilitate the entry of SARS-CoV-2 into cells (7), which ultimately lead to the replication of the virus and its release *via* exocytosis to infect more cells. While this ACE2-mediated SARS-CoV-2 cell entry is widely accepted, the mechanism underlying the progression of the virus infection and the heterogeneous severity (asymptomatic, lightly symptomatic to severe, critical, or even lethal conditions) (8) remains poorly understood. One important finding was that the asymptomatic individuals exhibit similar level of cytokines as the healthy ones, while the symptomatic individuals exhibit elevated level of cytokines (9). It is well-known now that COVID-19 patients under critical conditions experience hyperproduction of cytokines or so-called “cytokine storm” (10) *via* unknown factors that cannot be purely explained by the ACE2-dependent virus infection in the respiratory system. More importantly, studies have shown that SARS-CoV-2 only causes lung infection without severe disease progression in transgenic mice expressing human ACE2 (hACE2) under endogenous promoter, but the SARS-CoV-2 infection can spread to other organs in addition to lung in K18 transgenic mice expressing hACE2 under an ectopic cytokeratin promoter (11). These observations suggest that although ACE2 is responsible for the initial invasion of SARS-CoV-2, it is highly likely that there exists additional mechanisms for promoting further progression of SARS-CoV-2 infection. Some membrane proteins such as neuropilin-1 and CD147 present in ACE2-positive cells have been proposed to cooperate with ACE2 to regulate the virus infection (12), but whether and how SARS-CoV-2 infects non-ACE2 cells that may promote the progression of COVID-19 is unclear. Interestingly, recent studies using mouse models expressing hACE2 (13) and human COVID-19 patient samples (14, 15, 16) revealed that SARS-CoV-2 not only invades ACE2-positive cells but also non-ACE2 cells, suggesting the existence of unknown ACE2-independent entry mechanism for SARS-CoV-2. Bioinformatics and structure-based analyses revealed that SARS-CoV-2 RBD contains a surface-exposed arginine–glycine–aspartic acid (R^403^GD) motif that is absent in other coronaviruses (17, 18, 19). Such RGD motif is known to be recognized by several members of a family of cell adhesion receptors, integrins, which are (α/β) heterodimeric transmembrane proteins containing large extracellular domain, transmembrane domain, and small cytoplasmic tail (20). Integrins were previously shown to recognize RGD containing viruses, such as HIV, adenovirus, and foot-and-mouth disease virus, and mediate their cell entry *via* integrin-mediated endocytosis (21). Increasing computational and cellular studies have therefore been performed to examine whether integrins may engage SARS-CoV-2 and regulate the infectious life cycle of the virus (15, 17, 18, 22, 23, 24, 25, 26). However, while these studies provided important information of potential role of integrins in SARS-CoV-2 infection, no quantitative analysis has been performed so far to definitively evaluate the SARS-CoV-2 binding to integrin in comparison to ACE2. Moreover, the mechanism of how integrin acts on SARS-CoV-2 in relation to ACE2 remains highly elusive and controversial. For example, a significant number of studies proposed that integrin interacts with ACE2 to cooperatively promote the SARS-CoV-2 infection (15,17,18,19, 22,24,25,26), whereas some indicated that integrin inhibits the SARS-CoV-2 binding to ACE2 (23, 27). Also, one cell-based study suggested the requirement of integrin activation for mediating the SARS-CoV-2 infection (26) but other studies (18, 21, 22, 23, 24) did not show such requirement. The cell-based studies so far mostly relied on cells that contain both integrin and ACE2 (*e.g.*, VERO E6 cells), which might have contributed to the uncertainty of the integrin function. Furthermore, the use of the integrin antagonists, which have off-target effects and toxicity to cause cell death, may also partially contribute to the uncertainty of the studies.

Here, we use a combination of biochemical, biophysical, and cellular approaches with non-ACE2 cells (CHO-K1 cells) to investigate the role of the integrin-mediated cell entry of SARS-CoV-2. Using pull-down and surface plasmon resonance (SPR), we show that purified SARS-CoV-2 spike protein potently binds to widely distributed integrin α5β1 ectodomain with an affinity that is comparable to that between SARS-CoV-2 and ACE2. We further demonstrate that such binding leads to the internalization of SARS-CoV-2 into non-ACE2 cells in a manner that is critically dependent on the activation of integrin, its transition to a higher affinity/avidity state for cognate ligands. Our data thus provide proof-of-the-concept evidence for an alternative pathway of SARS-CoV-2 entry into cells. Given that initial SARS-CoV-2 infection *via* ACE2 in the respiratory system is known to generate cytokines (28) that can activate integrins (29, 30, 31), our data suggest that more SARS-CoV-2 particles may enter into (*via* the activated integrin) various human cells and tissues especially those lacking ACE2 thereby contributing to further infection and progression of COVID-19. Our findings may bear important implications in fighting against the global pandemic of COVID-19.

## Results

### Biochemical and biophysical analysis of SARS-CoV-2 binding to integrin α5β1

**Figure 1:**
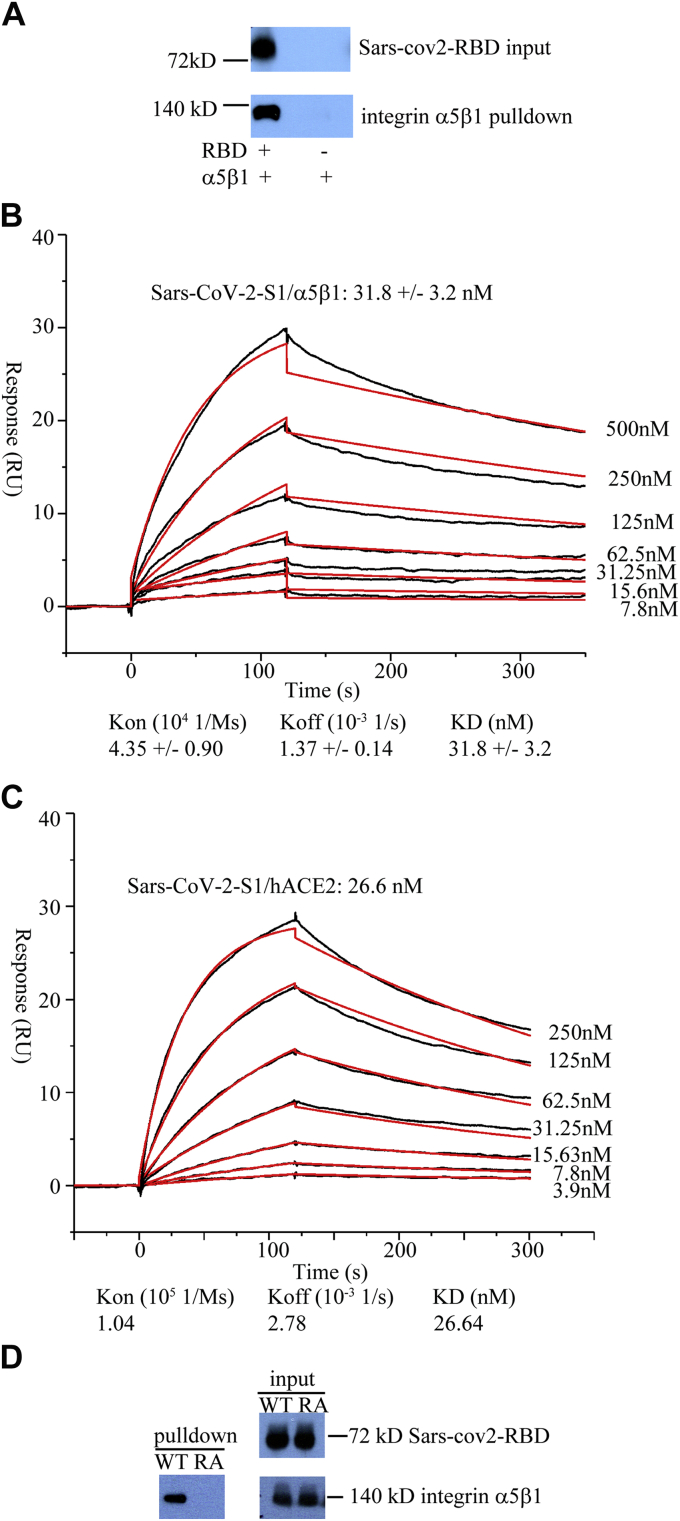
**SARS-CoV-2 spike protein interaction with integrin extracellular domain.***A*, biotinylated SARS-CoV-2-RBD is able to pull down integrin α5β1 (*left*) while there is no nonspecific binding of integrin to streptavidin beads (*right*) (Fig. S2). *B*, SPR affinity measurements of biotinylated SARS-CoV-2-S1 with integrin α5β1 extracellular domain. *Black curves* are SPR sensorgrams of various concentrations (7.8 nM, 15.6 nM, 31.25 nM, 62.5 nM, 125 nM, 250 nM, and 500 nM) of integrin flowed onto the chip surface immobilized with biotinylated SARS-CoV-2-S1. *Red curves* are 1:1 kinetics fitting, resulting in K_D_ ∼ 31 nM (n = 2). Low concentration point 3.9 nM had no response and was not used in the data fitting. Note that the fitting especially at high integrin concentration (500 nM) was not ideal likely due to the small degree (5–8%) of self-association (aggregation) and nonspecific binding of integrin despite the addition of BSA. This problem may somewhat affect the accuracy of K_D_. Removing the high concentration point (500 nM) made the fitting look better but does not significantly alter the binding kinetics; *C*, SPR affinity measurements of biotinylated SARS-CoV-2-S1 with hACE2 extracellular domain. *Black curves* are SPR sensorgrams of various concentrations (3.9 nM, 7.8 nM, 15.6 nM, 31.25 nM, 62.5 nM, 125 nM, and 250 nM) of hACE2 flowed onto the chip surface immobilized with biotinylated SARS-CoV-2-S1. Red curves are 1:1 kinetics binding fitting, resulting in K_D_ ∼ 26 nM. *D*, biotinylated SARS-CoV-2-RBD R403 A mutant (RA) is defective in binding to integrin α5β1 compared to its wild type (WT) counterpart (Fig. S3). Integrin α5β1 was detected using antibody specific to flag-tag attached to α5 subunit. ACE2, angiotensin-converting enzyme 2; hACE2, human ACE2; RBD, receptor binding domain; SARS-CoV-2, severe acute respiratory syndrome coronavirus 2; SPR, surface plasmon resonance.

As mentioned above, while numerous studies have indicated the binding of integrin to SARS-CoV-2 (15, 17, 19, 22, 23, 24, 25, 26, 27), no detailed biochemical and biophysical analysis has been performed to elucidate the binding event. To definitively detect the interaction between SARS-CoV-2 and integrin, we first performed the pull-down experiments using purified SARS-CoV-2 RBD and extracellular domain of well-known RGD-binding integrin α5β1, a fibronectin receptor that is broadly distributed, *e.g.*, in fibroblasts, endothelial cells, and blood cells. Figure 1*A* shows that SARS-CoV-2 RBD exhibits potent binding to α5β1. We then performed SPR experiments, which revealed the binding affinity K_D_ between SARS-CoV-2 RBD and integrin at ∼31 nM (Fig. 1*B*). This affinity is in the same range as that between SARS-CoV-2 spike protein and ACE2 (K_D_ ∼ 26 nM, Fig. 1*C*), which was reported before (32). Figure 1*D* shows that mutating R403 to alanine within RGD motif on the SARS-CoV-2 RBD abolished its binding to integrin, further demonstrating the specificity of the binding and the importance of R^403^GD motif in SARS-CoV-2 RBD in recognition of integrin.

### Activation of integrin on cell surface promotes its binding to SARS-CoV-2 RBD

**Figure 2:**
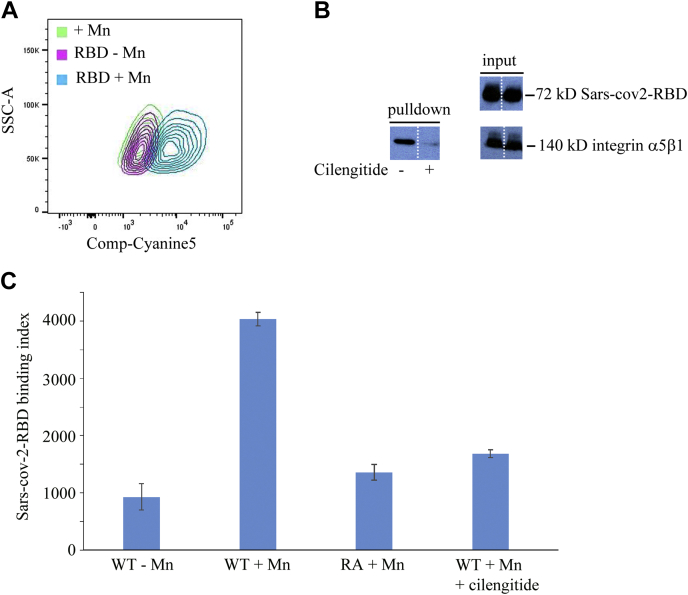
**SARS-CoV-2 RBD association with CHO-K1 cells requires activation of integrin.***A*, flow cytometry contour plots of CHO-K1 incubated with or without cyanine5 labeled SARS-CoV-2-RBD and with or without MnCl_2_. *B*, biotinylated SARS-CoV-2-RBD binding to integrin α5β1 is inhibited by Cilengitide. *Dashed lines* indicate spliced borders where data unrelated to the text were removed (see the original full blot in Fig. S4). *C*, bar graphs generated from flow cytometry data, which show that fixed CHO-K1 cells with activation of integrin by MnCl2 bind to cyanine5-labeled WT RBD, but the binding is substantially reduced upon R403A mutation or addition of integrin inhibitor Cilengitide. RBD, receptor binding domain; SARS-CoV-2, severe acute respiratory syndrome coronavirus 2.

To further examine the association of SARS-CoV-2 RBD with integrin, we next chose to use flow cytometry to detect the association of cyanine5-labeled RBD (Cy5-RBD) with CHO-K1 cells. CHO-K1 is a well-established cell line containing RGD-binding integrins, notably α5β1 (33, 34). The cell line was derived from Chinese Hamster Ovary and has no detectable ACE2 (5) let alone hACE2. In fact, the entire ovary has no detectable ACE2 (5). Thus, our assay avoids the complication of using cell lines that contain both integrin and ACE2 and is expected to gain definitive cellular evidence of the integrin–RBD interaction. However, first attempt to examine CHO-K1 treated by Cy5-RBD exhibited similar fluorescence intensity as untreated cells (data not shown), indicating that RBD has no significant association with integrins on the cell surface. Since integrins in unstimulated cells exist in predominantly inactive conformation that limits ligand access for binding (20), we stimulated the cells using MnCl2, a well-known integrin activating reagent (35, 36, 37, 38). The MnCl2 treatment substantially increased the fluoresce intensity induced by the association of Cy5-RBD with CHO-K1 cells (Fig. 2*A*), demonstrating that activation of integrin leads to robust binding to RBD on cell surface. This finding may explain why genetic screening failed to identify integrin as the SARS-CoV-2 host factor (39, 40, 41) since integrin in the screening assay may have been in an inactive state without agonist stimulation. We note that RBD interacted potently with purified integrin α5β1 ectodomain without Mn2+ treatment in our pull-down and SPR assays (Fig. 1). This is because in the absence of cellular constraints such as plasma membrane and integrin transmembrane-cytoplasmic portion known to control the inactive state of the receptor (20), the ectodomain can be readily converted from inactive to active state by ligand, resulting in total high affinity binding, *e.g.*, fibronectin repeats 9 to 10 can bind to α5β1 ectodomain at K_D_ ∼ 5.2 nM without any activating stimuli (42). This affinity is comparable to that of the fully activated (extension-open) intact α5β1 (K_D_ ∼ 1.4 nM) yet is >1730 times lower than inactive (bent-closed) intact α5β1 (K_D_ ∼ 9000 nM) (see Fig. 7E of Li *et al.*, (42)). The low ligand affinity of inactive receptor is clearly due to the restraint of cell membrane and transmembrane-cytoplasmic domain. At basal level (without any cellular stimulation), intact α5β1 exists in a dynamic equilibrium between inactive and active states, which binds fibronectin repeats 9 to 10 at K_D_ ∼ 1100 nM (stronger than the bent-closed intact receptor at K_D_ ∼ 9000 nM), which may explain why some previous studies (19, 22) detected the SARS-CoV-2 spike binding to integrin without cellular stimulation. Nevertheless, our study is consistent with a recent study showing that activation of integrin is crucial for its binding to UV-inactivated SARS-CoV-2 (26) and also with previous studies showing that activation of integrins is required for their binding to some large enveloped viruses such as HIV (37) but is not required for small non-enveloped virues such as foot-and-mouth disease virus (43) (SARS-CoV-2 is also enveloped virus).

To further confirm that the association of RBD with CHO-K1 cells is dependent on integrin but not on other receptors such as neuropilin-1, CD147, heparin sulfate, sialic acid, or a number of other molecules that the spike protein has been reported to bind, we examined the RBD binding efficiency using R403A mutation to disrupt the RGD motif and also Cilengitide—a RGD analog cyclic pentapeptide that can potently inhibit binding of RGD ligands to αvβ3 (IC_50_ ∼ 0.61 nM), αvβ5 (IC_50_ ∼ 8.4 nM), and α5β1 (IC_50_ ∼ 14.9 nM), respectively (44). Figure 2*B* illustrates Cilengitide effectively inhibited the RBD binding to the purified integrin α5β1 extracellular domain. However, when we attempted to examine the RBD/integrin binding efficiency using flow cytometry in the presence of Mn^2+^, neither R403A mutation nor inhibitor had any significant effect (data not shown). Shayakhmetov *et al.* (45) previously reported that RGD motif deletion in adenovirus did not significantly affect the virus attachment but reduced the virus internalization. This led us to consider if RBD is rapidly internalized upon binding to the Mn^2+^-activated integrin, which compensated the binding defects from R403A and inhibitor treatment. To circumvent this problem, we modified the cell preparation protocol by fixing cells first before incubating with RBD protein. In this way, we only look at the protein attachment while excluding the potential internalization of RBD. Figure 2*C* shows that R403A mutation dramatically reduced association of RBD with CHO-K1 cells, and Cilengitide also significantly inhibited RBD association with CHO cells, demonstrating that RBD specifically binds to active integrin on the cell surface.

### Internalization of SARS-CoV-2 RBD by activated integrin α5β1

**Figure 3:**
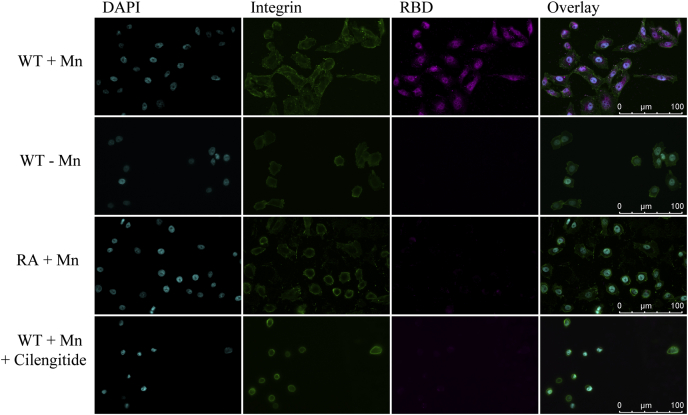
**SARS-CoV-2-RBD is able to internalize into CHO-K1 cells upon integrin is activated, and the internalization is dependent on RGD motif.** The *first row*, cyanine5-labeled SARS-CoV-2-RBD WT shown in *magenta* is able to internalize into CHO-K1 cells in the presence of MnCl_2_. The *second row*, cyanine5-labeled SARS-CoV-2-RBD WT is unable to internalize into CHO-K1 cells in the absence of MnCl_2_. The *third row*, cyanine5-labeled SARS-CoV-2-RBD R403A mutant (RA) is unable to internalize into CHO-K1 cells in the presence of MnCl_2_. The *fourth row*, cyanine5-labeled SARS-CoV-2-RBD WT internalization into CHO-K1 cells in the presence of MnCl_2_ is impaired by Cilengitide. RBD, receptor binding domain; R^403^GD, arginine–glycine–aspartic acid; SARS-CoV-2, severe acute respiratory syndrome coronavirus 2.

Next, we wanted to experimentally examine if SARS-CoV-2 RBD can indeed internalize into cells by binding to integrin α5β1 as we speculated. If so, this would suggest that SARS-CoV-2 can enter non-ACE2 cells through the route of integrin endocytosis. Integrin is well known to quickly internalize soluble ligands into endosomes *via* endocytosis (21, 46). Integrin-bound ligand can be also internalized but may dissociate from integrin and gradually degrade in lysosomes while free integrin can recycle back to the cell membrane (47). Although the membrane fusion is deemed to mediate the cell entry of SARS-CoV *via* ACE2 route, recent evidence showed that SARS-CoV can also be internalized *via* an endocytosis route (48). It is thus possible that viruses take advantage of routes of membrane fusion and/or endocytosis, depending on which route is available. In support of our speculation, Figure 3 shows that RBD can indeed be internalized into Mn^2+^-treated cells but not in cells without the Mn^2+^ treatment. Thus, activation of integrin is required for effective internalization of RBD into cells, which is consistent with the above data of RBD binding to activated integrin α5β1 (Fig. 2*A*). We also stained integrin β1 to mark the cell boundary without disruption of the cell membrane. Importantly, R403A mutation substantially reduced the internalization, indicating that RBD is specifically internalized through the association with the integrin (Fig. 3). We also treated cells with Cilengitide, and our results show that Cilengitide can significantly inhibit RBD internalization. Our results thus provide strong evidence that SARS-CoV-2 RBD can internalize into cells through binding to activated integrin α5β1 in ACE2-indepenent manner. As mentioned earlier, several viruses such as human adenovirus, foot-and-mouth disease virus, human herpes viruses, etc. were shown to internalize into cells *via* integrin endocytosis (21) and thus integrin α5β1 highly likely uses the same endocytosis route to internalize SARS-CoV-2.

### Cell entry of SARS-CoV-2 through activated integrin α5β1

**Figure 4:**
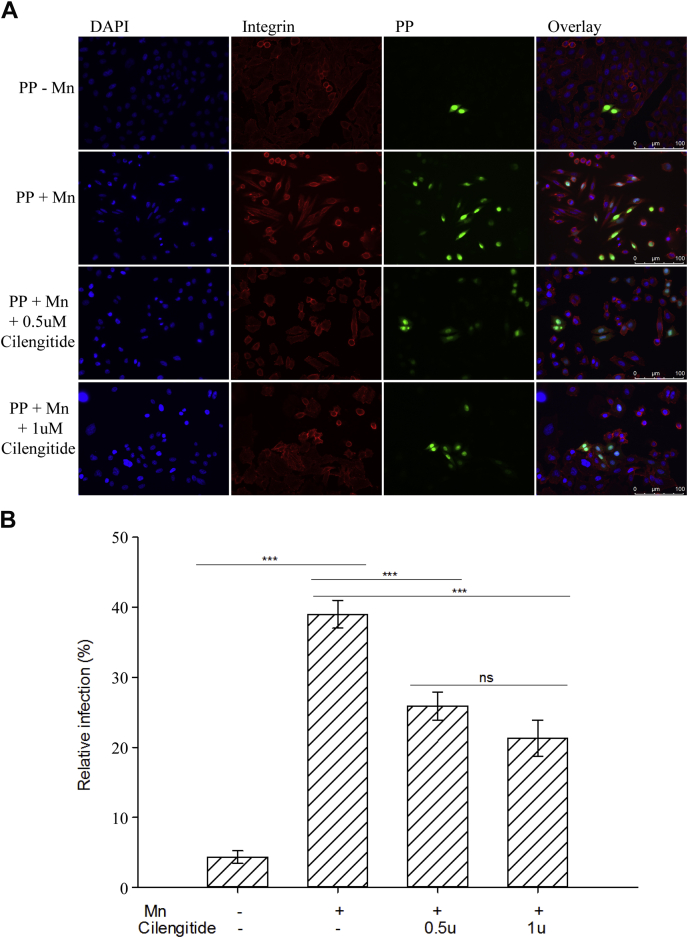
**SARS-CoV-2 pseudovirus infection to CHO-K1 cells.***A*, representative images of SARS-CoV-2 pseudovirus particle (PP) infection on CHO-K1 cells upon integrin activation by MnCl_2_ and the inhibition by integrin inhibitor Cilengitide. The *first row*, SARS-CoV-2 PP barely infects CHO-K1 cells in the absence of MnCl_2_. The *second row*, SARS-CoV-2 PP massively infects CHO-K1 cells in the presence of MnCl_2_. The *third* and *fourth rows*, SARS-CoV-2 PP infection on CHO-K1 cells in the presence of MnCl_2_ is impaired by Cilengitide. *B*, quantification of the relative infection of three randomly selected regions from each of three independent experiments. Data are averages ± SEM for nine images (three randomly selected images from each three independent experiments). ∗∗∗*p* < 0.001. ns, not significant, *p* > 0.05. SARS-CoV-2, severe acute respiratory syndrome coronavirus 2.

Ideally, the cellular entry of SARS-CoV-2 *via* integrin needs to be tested using a live virus. Because we do not have access to BSL3 laboratory, we chose to use the live pseudovirus, a well-recognized and reliable tool to examine early events of virus infection such as virus attachment and cell entry. Pseudoviruses have also been used to generate several COVID-19 vaccines such as AstraZeneca COVID-19 vaccine that is a modified adenovirus. Here we used baculoviruses pseudotyped with SARS-CoV-2 spike proteins (Bozeman; cat.no. C1110G) (49, 50, 51) to infect CHO-K1 cells and hACE2-expressing CHO-K1 cells. Once entering cells, this pseudovirus delivers a mNeonGreen reporter gene that expresses the bright green fluorescent protein in the host cell nucleus. After virus infection, we also stained cells with integrin β1 antibody to show the cell contour. Figure 4 shows that the pseudovirus clearly enters CHO-K1 cells in the absence of hACE2, and the cell entry is critically dependent on the activation of integrin by Mn^2+^. Forty percent of cells were infected within 24 h incubation, although a longer incubation time can lead to even higher infection rate (data not shown). Furthermore, the entry of the pseudovirus was inhibited by Cilengitide (infection rate reduced to ∼20%). These data, combined with the above RBD-based cellular entry data, demonstrate that the cell entry of SARS-CoV-2 can be mediated by activated integrin in non-ACE2 cells, clearly independent of ACE2, or other molecules that the spike protein has been reported to bind. To our knowledge, this is the first direct evidence showing the integrin-dependent cell entry of the SARS-CoV-2 in non-ACE2 cells.

**Figure 5:**
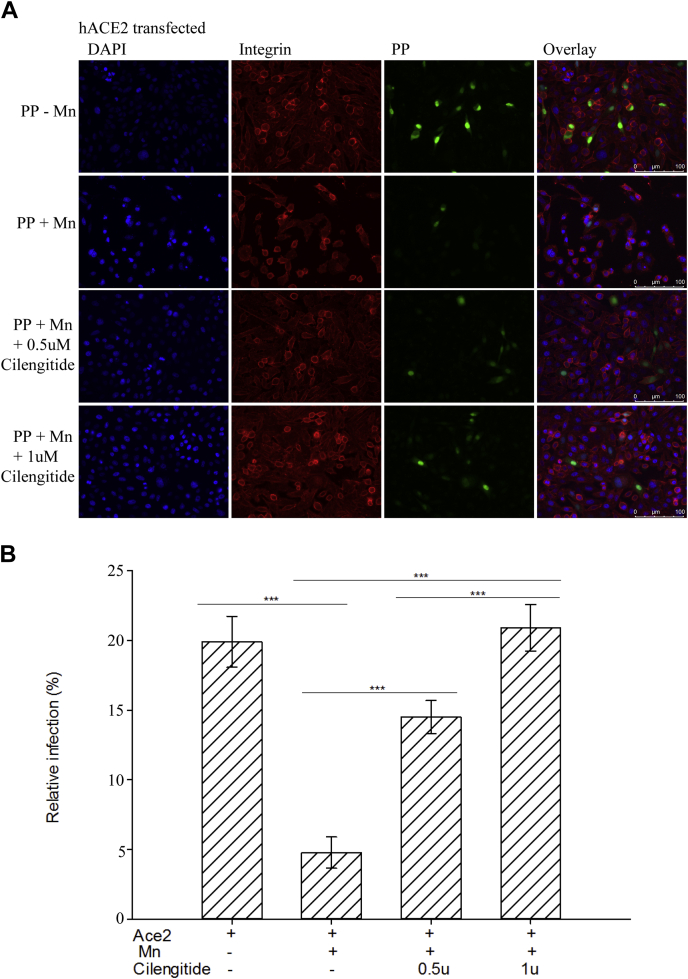
**Integrin and ACE2 are independent receptors for SARS-CoV-2 pseudovirus infection on hACE2 transiently transfected CHO-K1 cells.***A*, representative images of SARS-CoV-2 pseudovirus particle (PP) infection on hACE2 transiently transfected CHO-K1 cells upon integrin activation by MnCl_2_ and the inhibition of integrin by inhibitor Cilengitide. The *first row*, SARS-CoV-2 PP massively infects hACE2 transiently transfected CHO-K1 cells in the absence of MnCl_2_. The *second row*, SARS-CoV-2 PP barely infects hACE2 transiently transfected CHO-K1 cells upon integrin activation in the presence of MnCl_2_. The *third* and *fourth rows*, SARS-CoV-2 PP infection on hACE2 transiently transfected CHO-K1 cells upon integrin activation in the presence of MnCl_2_ is gradually recovered by Cilengitide. *B*, quantification of the relative infection of three randomly selected regions from each of three independent experiments. Data are averages ± SEM for nine images (three randomly selected images from each three independent experiments). ∗∗∗*p* < 0.001. Note: the infect rate here is lower than Figure 4, which is because hACE2 here is transiently transfected while integrin is endogenously expressed. ACE2, angiotensin-converting enzyme 2; hACE2, human ACE2; SARS-CoV-2, severe acute respiratory syndrome coronavirus 2.

**Supplementary Figure S1:**
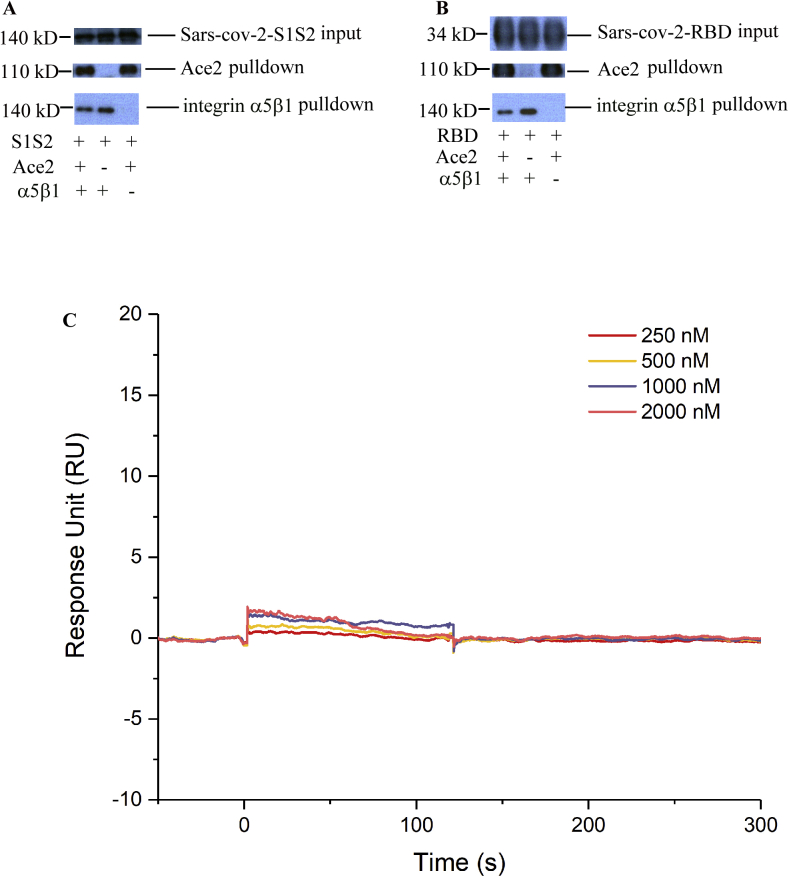
SARS-CoV-2 relationship with integrin and ACE2. *A*, biotinylated SARS-CoV-2-S1S1 or (*B*) biotinylated SARS-CoV-2-RBD (SINO BIOLOGICAL #40592-V08B-B) is able to pull down integrin α5β1 in the presence or absence of hACE2 (Figs. S5S6). The hACE2 slightly reduced the binding of SARS-CoV-2 RBD to integrin probably because the binding sites of hACE2 and integrin on RBD are spatially close and thus the two proteins sterically clash when both bind to RBD. *C*, SPR experiment showing no significant interaction between immobilized biotinylated ACE2 (SINO BIOLOGICAL #10108-H27B-B) and integrin α5β1.

To gain insight whether integrin can act independently or cooperate with ACE2 to mediate the SARS-CoV-2 entry, we expressed hACE2 in CHO-K1 cells. In comparison with Figure 4 where CHO-K1 had low level of SARS-CoV-2 entry without integrin activation by Mn^2+^ treatment, Figure 5 shows that expression of hACE2 induced the high level of SARS-CoV-2 entry, indicating that hACE2 can effectively mediate the cell entry of SARS-CoV-2. Interestingly, upon Mn^2+^ treatment to activate integrin, the SARS-CoV-2 entry was dramatically impaired in the hACE2-expressing CHO-K1 cells, whereas the inhibition of integrin by Cilengitide can gradually recover the cell entry of the virus apparently through the hACE2 route (the integrin route is suppressed by Cilengitide) (Fig. 5). These results indicate that the two entry receptors do not cooperate with each other. Rather, they are independent and mutually exclusive from each other when both are expressed on the same cell surface with integrin being also activated. Pull-down experiments reveal that integrin could still bind to SARS-CoV-2 spike protein or RBD in the presence of ACE2, albeit with reduced capacity (Fig. S1, *A* and *B*) probably due to the steric clash between integrin and ACE2 when binding to the same SARS-CoV-2 since RGD site is spatially close to the ACE2 site on RBD (17, 27). There is no detectable interaction between integrin and ACE2 (Fig. S1*C*), ruling out the possibility that the two receptors either inhibit or cooperate with each other *via* direct interaction. However, ACE2 and activated integrin likely bind to different spike proteins of the same SARS-CoV-2 virus. Because ACE2 (7) and integrin (20, 44) use different routes and kinetics to mediate the virus entry, such simultaneous binding may inhibit the virus entry as seen in Figure 5 since the same virus cannot enter the cell *via* two different doors/routes at the same time. Interestingly, some human cancer cells naturally containing both ACE2 and integrin were also found to be resistant to SARS-CoV-2 (Fig. 1D of Puray-Chavezet *et al.*, (52)). It was also reported (23) that integrin αvβ5 and hACE2 expressed on 293T cells inhibit each other for the virus binding and infection. These studies contrast to the data obtained from VERO cells that were found to be permissive to the SARS-CoV-2 infection and inhibited by integrin inhibitors (19, 26). Thus, it appears that different human cells respond differently to SARS-CoV-2 depending on conditions that are not fully understood. The expression levels of ACE2 and integrin and their ratio in cells containing both receptors may play a critical role in regulating the virus infection. The complete mechanism as to why and how cells containing both ACE2 and integrin respond differently to SARS-CoV-2 remains to be further investigated. Nevertheless, our studies using non-ACE2 cells demonstrate clearly that integrin α5β1 functions independently of ACE2 to mediate the SARS-CoV-2 internalization.

## Discussion

Although SARS-CoV-2 has been intensively studied ever since the global pandemic started, its high contagiousness and high hospitalization/mortality rate compared to seasonal influenza still remain poorly understood. The infection clearly started locally in upper respiratory system but how it progresses further to severe, critical, or even lethal medical conditions remains enigmatic. Using multiple independent methods, we provide strong evidence that in addition to binding to well-known receptor ACE2, SARS-CoV-2 spike protein also associates with integrin with an affinity that is comparable to that between SARS-CoV-2 and ACE2. More importantly, we demonstrate that SARS-CoV-2 invades cells upon binding to activated integrin α5β1, and such infection is ACE2 independent. The infection appears to be also independent from other molecules that the spike protein has been reported to bind because we used the integrin-specific RGD mutation, inhibitor, and Mn^2+^ treatment. The requirement of integrin activation to bind to SARS-CoV-2 explains why previous genetic screening failed to identify integrin as the SARS-CoV-2 host factor (39, 40, 41), since integrin in the screening assay is apparently in inactive state without agonist stimulation. Our findings suggest that while ACE2-mediated SARS-CoV-2 invasion plays a crucial role in initiating the infection in the respiratory system, activation of RGD binding integrins such as α5β1 may further contribute to deeper infection by binding and internalizing virus into more cells and tissues especially those non-ACE2 containing ones. The latter is consistent with the detections of SARS-CoV-2 in non-ACE2 cells of mouse model (13), lung (52), brain (14), and tissue samples of COVID-19 patients (15, 16). A key question is when and how are integrins activated to mediate the SARS-CoV-2 infection in connection with the ACE2 pathway? As we mentioned earlier, although ubiquitously expressed, integrins are highly controlled receptors that are typically in the inactivated conformation with a very low ligand binding affinity. The control of integrin inactivation is likely even tighter for epithelial cell integrins that are exposed to the external environment such as in the upper respiratory system. Even if there exist a small population of active integrins, they might be tightly bound to natural ligands and unavailable for virus binding. To activate integrin, a cascade of cellular signals triggered by cytokines (29, 30, 31) or other stimuli may impinge on integrin cytoplasmic tails causing inside-out conformational change of the receptor (20). Such feature makes integrin an inferior entry receptor for virus. On the other hand, although the expression of ACE2 is low and restricted in certain organs, especially in the respiratory system exposed to the external environment, ACE2 is constantly available to bind the viral ligand. Based on this scenario and our results, we speculate two sequential events leading to the progression of COVIC-19: (i) SARS-CoV-2 first recognizes ACE2 in the upper respiratory system to trigger the initial virus infection. (ii) The ACE2-initiated SARS-CoV-2 infection induces inflammation and generation of cytokines released to the respiratory system and subsequently other parts of the body, which in turn trigger signals to activate RGD binding integrins on many cells especially those non-ACE2 cells, thus causing more entry and massive replication of the virus in various tissues and organs, which ultimately leads to the deterioration of the clinical status such as severe infection and multiorgan failure. Such combination of ACE2 and integrin-mediated virus spreading is likely one important mechanism to attribute to high contagiousness and high hospitalization/mortality rate of SARS-CoV-2 as summarized in the following disease progression: Initial ACE2 cell infection → cytokine release → integrin activation → non-ACE2 cell infection → more cytokine release → more integrin activation → more non-ACE2 cell infection → cytokine storm → severe symptoms/mortality. This scenario is reminiscence of HIV (human immunodeficiency virus) infections that firstly lure the immune response and then attack the immune cells. Thus, given the analysis of asymptomatic individuals (9) and the comparison of transgenic mice expressing hACE2 under the endogenous *versus* ectopic promoters (11), as well as our results, inhibiting the integrin activation may significantly reduce the SARS-CoV-2 infection, especially the severe infection-induced hospitalization/mortality rate.

## Experimental procedures

### SARS-CoV-2 RBD purification and labeling

SARS-CoV-2 RBD cDNA (T333-K529) was cloned into PHL-mMBP-10 vector (Addgene #72348). MBP-tagged RBD protein was produced from HEK293 T cells. The cells were seeded in Dulbecco's modified Eagle's medium (DMEM) supplemented with 10% fetal bovine serum (FBS) for overnight. When cells grew to 70 to 80% confluency, plasmid DNA was transfected using JetOPTIMUS transfection reagent (Polyplus). After 4 h, medium was replaced by fresh DMEM without FBS. Conditioned medium containing secreted mMBP-RBD was harvested 3 days after transfection. Conditioned medium was adjusted to 10 mM imidazole, 150 mM NaCl, and 20 mM Hepes pH 8.0 (binding buffer). Pre-equilibrated NI-NTA agarose slurry (1 ml) (Thermofisher) was added and allowed to incubate overnight at 4 °C using a rotary shaker. Beads were collected and washed with binding buffer plus extra 10 mM imidazole. Proteins were eluted with binding buffer plus 350 mM imidazole. Elution fractions were concentrated and subjected to superdex 200 size-exclusion chromatography pre-equilibrated with 100 mM NaCl, 20 mM Hepes pH 8.0 for purification. R403A mutation was made by in-fusion cloning kit (Takara Bio). Biotinylated RBD and RA RBD were generated using EZ-link Sulfo-NHS-Biotin kit (Thermofisher). Cy5-labeled RBD was made with Cyanine5 NHS ester (Lumiprobe).

### Pull-down and Western blot

Biotinylated SARS-CoV-2 S1, biotinylated SARS-CoV-2 S1S2, biotinylated SARS-CoV-2 RBD (#40592-V08H-B), hACE2-his tag (#10108-H08B), and integrin α5β1 extracellular domain with flag tag on α5 and His tag on β1 (#CT014-H2508H) were purchased from SINO BIOLOGICAL, Inc The same amount of biotinylated proteins was loaded on 20 μl Streptavidin beads (Cell signaling #3419) prewashed with Hepes buffer (20 mM Hepes, 200 mM NaCl, 1 mM CaCl_2_, 0.05% Tween20, 0.05% NaN_3_, PH 7.4). The beads were incubated for 30 min at 4 °C and washed with 10 volume of Hepes buffer. The same amount of integrin was added into beads and incubated for overnight at 4 °C. Beads were collected and washed with 10 volume of Hepes buffer, then resuspended in SDS loading dye, and loaded onto SDS gel. The integrin α5β1 was detected by anti-flag antibody (Sigma-Aldrich #F1804). ACE2 was detected by his-tag antibody (Novagen #70796), and biotinylated proteins were detected by biotin antibody (Cell signaling #7075).

### SPR experiments

SPR experiments were carried out on the BIAcore T200 (Cytiva, former GE Healthcare) at Case Western Reserve University Protein Expression Purification Crystallization and Molecular Biophysics Core. Biotinylated SARS-CoV-2-S1 (SINO BIOLOGICAL #40591-V08H-B) was immobilized onto streptavidin chip. The kinetic assay was run at 25 °C and in 20 mM Hepes, 200 mM NaCl, 1 mM CaCl_2_, 0.05% Tween20, 0.03% BSA, 0.05% NaN_3_, PH 7.4. Results were analyzed by Biacore T200 Evaluation software 3.1 using 1:1 kinetics binding model and redrawn by Origin 7.0.

### Flow cytometry analysis

About 80% confluent CHO-K1 cells were harvested with nonenzyme disassociation solution (Corning) and were resuspended at 1 × 10^7^ cells per ml in Hanks' banlanced salt solution (HBSS, Thermofisher, Gibco, #140525-076) plus 5% BSA in the presence and absence of 1 mM manganese chloride, in the presence and absence of Cilengitide (Sigma, SML 1594-5MG) for 30 min at room temperature. Note that the nonenzyme disassociation solution differs from the trypin used in ref 26 that is known to cleave membrane proteins. Cells were centrifuged at 500*g* for 5 min after washing and fixed in 2% paraformaldehyde (PFA) in phosphate-buffered saline. Cells were washed again and treated with 5 μg cy5-labeled RBD per 1 × 10^6^ cells in 100 μl for 30 min at room temperature. Cells were washed and resuspended in HBSS. The association of RBD with CHO cells was analyzed by LSRFortessa Cell Analyzer (BD) in Cleveland Clinic Lerner Research Institute Flow Cytometry core. For live cell treatment for RBD binding, cells were first incubated with cy5-labeled RBD in the presence and absence of 1 mM manganese chloride and in the presence and absence of Cilengitide for 30 min at room temperature. Cells were then washed and fixed in 2% PFA. Live cells data were not shown.

### Internalization assay

Briefly, 1.5 × 10^5^ CHO-K1 cells (ATCC CCL-61) were seeded on a microscope coverslips (d = 18 mm, Fisherbrand) within 12-well plate with 1 ml complete medium (DMEM:F12 supplemented with 10% FBS). Cells were incubated under normal growth conditions (37 °C and 5% CO2) for overnight. CHO-K1 cells would reach about 80% confluency the next day, and they were cooled down on ice for 5 min first. Media were then removed and replaced with 400 μl cold buffers containing 12 μg cy5-labeled RBD protein and integrin primary antibody (7E2 Developmental Studies Hybridoma Bank) in HBSS with 5% BSA in the presence and absence of 1 mM manganese chloride, in the presence or absence of Cilengitide. Cells were incubated on ice for 1 h and incubated at 37 °C for another 30 min. After the incubation, cells were cooled down on ice for 5 min and then washed with cold HBSS, fixed with 4% formaldehyde for 20 min at room temperature. Cells were washed with HBSS and then incubated with the secondary antibody (Goat anti-mouse IgG H&L Alexa 488, Abcam, #ab150117). Cells were washed and sealed on microscope slides (25 × 75 mm, Fisherfinest) with Diamond Antifade mountant with DAPI. Cells were visualized in Cleveland Clinic Lerner Research Institute Light Microscopy core under widefield upright Microscope at 40x magnification using the LAS X software.

### SARS-CoV-2 pseudovirus assays

SARS-CoV-2 Green reporter pseudovirus (Bozeman; cat.no. C1110G) was used according to the manufacturer instructions with minor modification under an approved protocol by Cleveland Clinic Institutional Biosafety Committee. Briefly, 1.5 × 10^5^ CHO-K1 cells (ATCC CCL-61) were seeded on a microscope coverslips (d = 18 mm, Fisherbrand) within 12-well plate with 1 ml complete medium (DMEM:F12 supplemented with 10% FBS). Cells were incubated under normal growth conditions (37 °C and 5% CO2) for overnight. hACE2 (Addgene #1786) was transfected using JetOPTIMUS transfection reagent (Polyplus) when overnight cell culture grew up to 50 to 70% confluency. Transfected cells were maintained for another 24 h. Then medium was removed, cells were washed once with PBS, and replaced with 0.5 ml fresh medium. A 0.5 ml transduction mixture of 25ul of 2 × 10^10^ VG/ml pseudovirus stock, supplemented with 4 mM valproic acid, with and without 1 mM Manganese Chloride, with and without Cilengitide, was added onto cells. The plate was centrifuged at 50*g* for 15 min at room temperature. Then, cells were incubated at 37 °C for 3 h, followed by replacing transduction mixture with fresh media. Cells were further maintained at 37 °C for 12 to 24 h. Cells were washed with HBSS+5%BSA, fixed with 4% PFA, stained with integrin β1 antibody (7E2 Developmental Studies Hybridoma Bank) followed by secondary antibody (Goat anti-mouse IgG Alexa 568, Invitrogen, #A11004) and sealed on microscope slides (25 × 75 mm, Fisherfinest) with Prolong Diamond Antifade mountant with DAPI. Cells were visualized by Gauravi Deshpande PhD at Cleveland Clinic Lerner Research Institute imaging core under widefield upright Microscope at 40x magnification using LAS X software. This assay was repeated three times, and three randomly selected areas per coverslip were imaged, which made nine images for each group. Briefly, to measure the relative infection, pseudovirus-positive and DAPI-positive cell numbers were quantified using Image-Pro plus 7.0. All the cells were counted with the same threshold setting for all images from each independent experiment. The percentage of pseudovirus infection was determined by dividing the number of pseudovirus positive cells by the number of DAPI positive cells.

## Data availability

All the relevant data are within the manuscript and its supporting information files.

## Supporting information

This article contains supporting information

## Conflict of interest

The authors declare that they have no conflicts of interest with the contents of this article.

## Figures and Tables

**Supplementary Figure S2:**
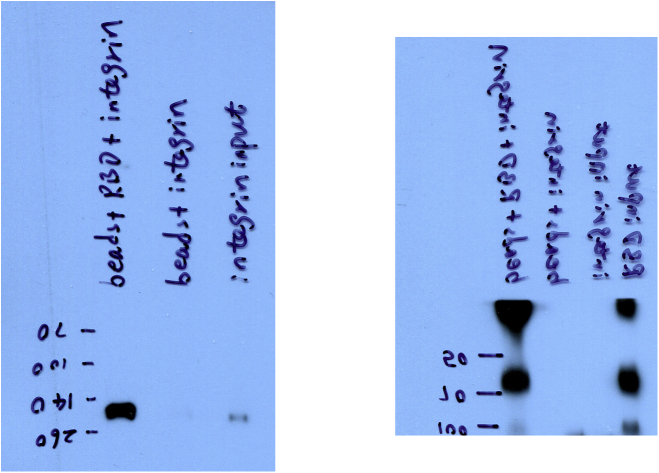
Unprocessed blots for Figure 1*A*.

**Supplementary Figure S3:**
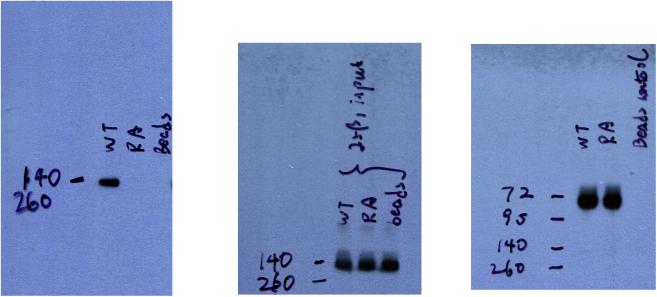
Unprocessed blots for Figure 1*D*.

**Supplementary Figure S4:**
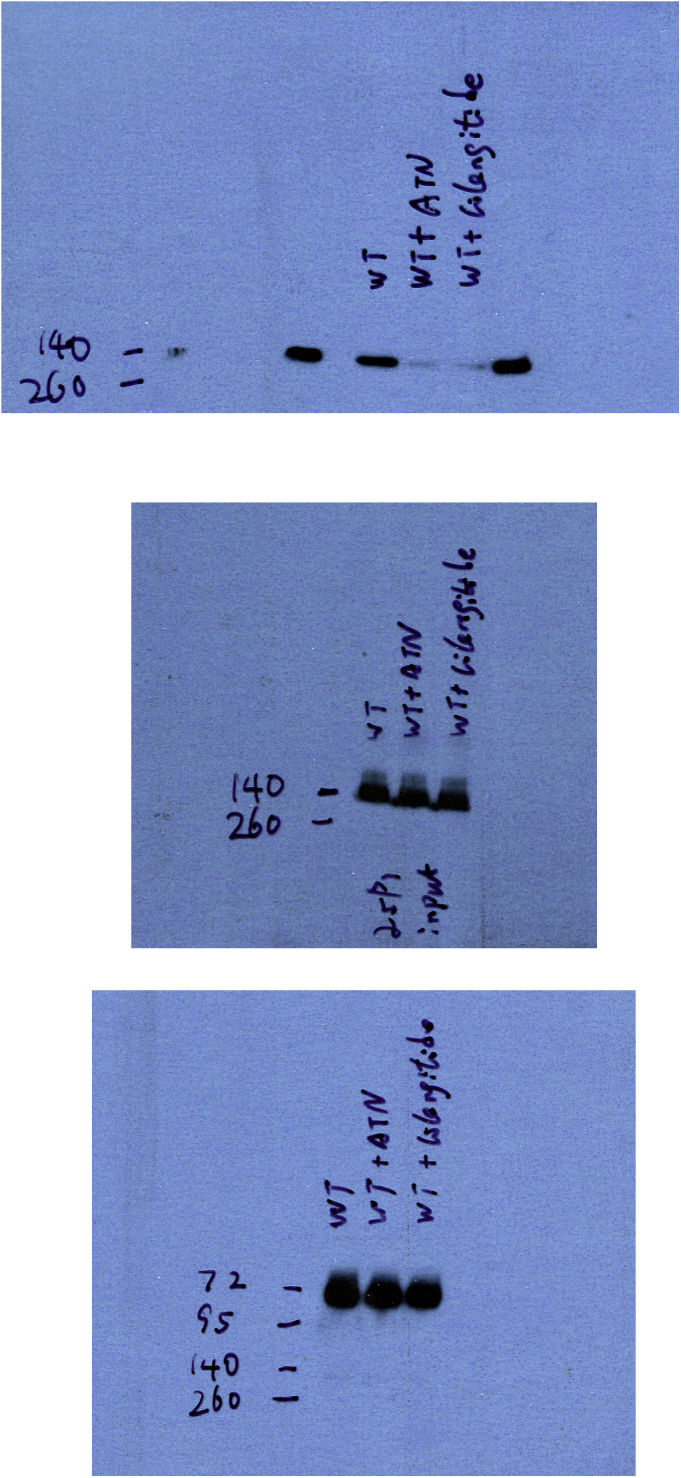
Unprocessed blots for Figure 2*B*.

**Supplementary Figure S5:**
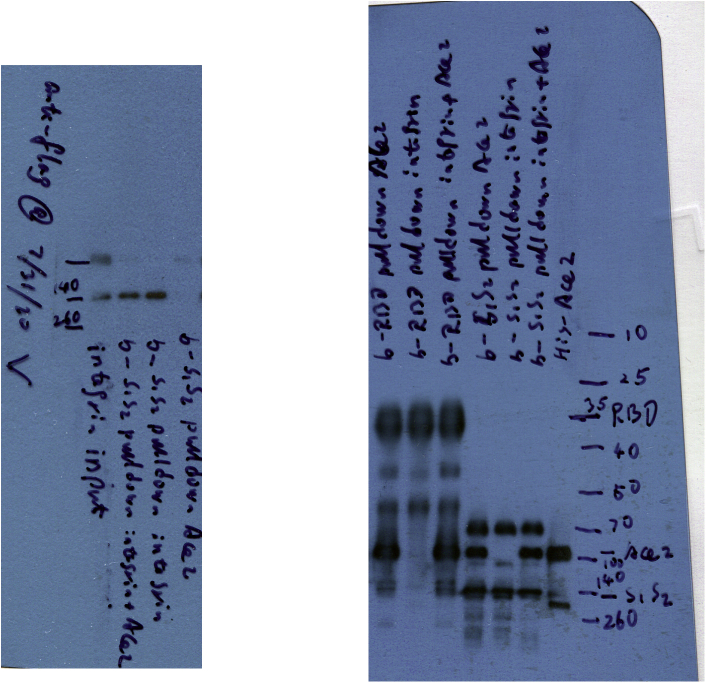
Unprocessed blots for Fig. S1*A*.

**Supplementary Figure S6:**
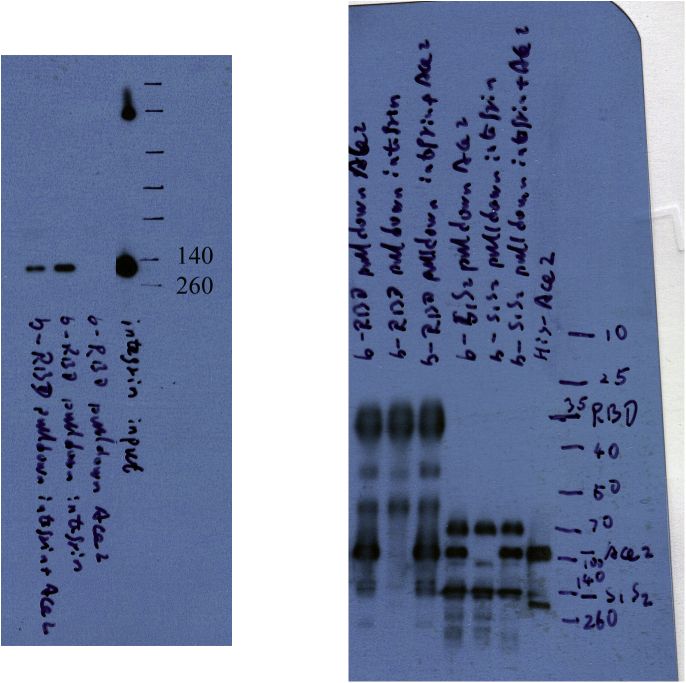
Unprocessed blots for Fig. S1*B*.
